# A High-Temperature Fiber Sensor Using a Low Cost Interrogation Scheme

**DOI:** 10.3390/s130911653

**Published:** 2013-09-04

**Authors:** David Barrera, Salvador Sales

**Affiliations:** Optical and Quantum Communications Group, Institute of Telecommunications and Multimedia Applications (iTEAM), Universitat Politècnica de València, Valencia, 46022, Spain; E-Mail: ssales@dcom.upv.es

**Keywords:** optical fibre sensor, high-temperature, Regenerated Fibre Bragg Grating, interrogation, modal interferometer

## Abstract

Regenerated Fibre Bragg Gratings have the potential for high-temperature monitoring. In this paper, the inscription of Fibre Bragg Gratings (FBGs) and the later regeneration process to obtain Regenerated Fiber Bragg Gratings (RFBGs) in high-birefringence optical fiber is reported. The obtained RFBGs show two Bragg resonances corresponding to the slow and fast axis that are characterized in temperature terms. As the temperature increases the separation between the two Bragg resonances is reduced, which can be used for low cost interrogation. The proposed interrogation setup is based in the use of optical filters in order to convert the wavelength shift of each of the Bragg resonances into optical power changes. The design of the optical filters is also studied in this article. In first place, the ideal filter is calculated using a recursive method and defining the boundary conditions. This ideal filter linearizes the output of the interrogation setup but is limited by the large wavelength shift of the RFBG with temperature and the maximum attenuation. The response of modal interferometers as optical filters is also analyzed. They can be easily tuned shifting the optical spectrum. The output of the proposed interrogation scheme is simulated in these conditions improving the sensitivity.

## 1. Introduction

Optical fibre sensors offer a good performance under extreme conditions because they have small dimensions and low weight, they are immune to electromagnetic interference, chemically inert and spark free [1]. Among the various types of optical fibre sensors, Fibre Bragg Gratings (FBGs) offer greater multiplexing capabilities for multipoint measurement and the fluctuations of the received power do not affect the measurements. However, for high-temperature monitoring FBGs present a progressive decay, limiting the range of operation [2]. Several techniques have been proposed to extend the operating temperature range of the FBGs: modifications of the composition of the fibre, inscription of Type II gratings and high-temperature annealing [3–5]. Regenerated Fiber Bragg Gratings (RFBGs) are obtained from a seed FBG after a high-temperature annealing process. During the annealing process, the reflected optical power of the seed FBGs decays, followed by an increase. This is known as the regeneration process, and gives rise to a RFBG with improved temperature stability. In the last years, the regeneration process has been studied under different conditions and with several optical fibres [6–10].

In this paper the fabrication of RFBGs in high-birefringence optical fibres is shown. The optical spectrum shows two resonances corresponding to the fast and slow polarizations which show a different wavelength shift with temperature. This effect is going to be used to reduce the complexity and cost of the interrogation of the high-temperature sensor. The proposed interrogation setup is based on the use of optical filters in order to convert the wavelength shifts of each of the polarizations into changes in the optical power. The optical filters have a significant influence in the output of the interrogation setup and their design is also addressed. The paper is organized as follows: Section 2 details the fabrication process of the RFBG in high-birefringence optical fibre. Section 3 presents the proposed interrogation setup and the design of the ideal filter. In Section 4, the use of modal interferometers as the optical filters is discussed. Finally, concluding remarks are provided.

## 2. RFBG Regeneration

A Fibercore HB1500T high-birefringence optical fibre is used. The core of the optical fibre is flanked by areas of high-expansion, boron-doped glass that shrink-back more than the surrounding silica as the fibre is drawn and freeze the core in tension. This tension induces birefringence and when a FBG is inscribed originates two grating peaks at different wavelength for the slow and fast axis. The difference in the thermal expansion coefficient of the boron-doped areas makes that the wavelength separation of the two Bragg resonances changes with temperature.

In order to be able to measure high-temperatures a RFBG is needed. In first place the optical fibre is introduced in a hydrogen loading chamber. The seed FBGs is then inscribed in the optical fibre using an argon-ion frequency doubled laser at 244 nm and a phase mask technique. Finally, the FBG is introduced in a tubular oven and a high-temperature annealing is performed. The optical spectrum is continuously monitored using during the annealing process by a Micron Optics SM125 FBG interrogator.

**Figure 1.**
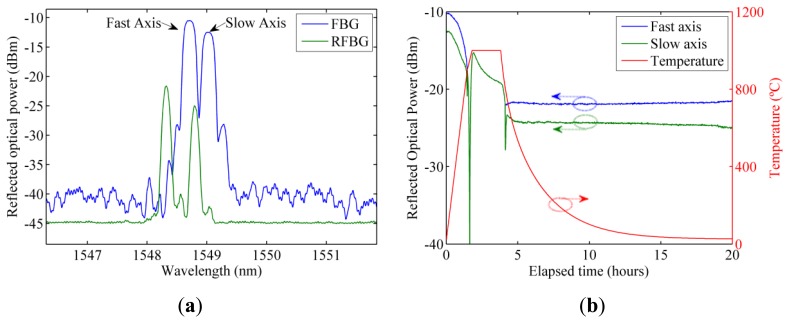
(**a**) Spectra of the seed FBG and RFBG; (**b**) Maximum reflected optical power of the fast and slow axis during the regeneration process.

Figure 1a shows the optical spectrum of one of the seed gratings and the RFBG obtained after the regeneration process described in Figure 1b. The regeneration process consist in a 10 °C/min temperature ramp up to 900 °C followed by a 5 °C/min temperature ramp up to 1,000 °C where the temperature is stabilized for 2 h. After this stabilization period, the oven is allowed to return to ambient temperature. It can be noticed the two resonances corresponding to the fast and slow polarizations. At the same temperature, the obtained RFBGs present a permanent wavelength shift and have a reduced reflectivity compared to the original FBG but the dynamic range is still around 20 dB.

**Figure 2.**
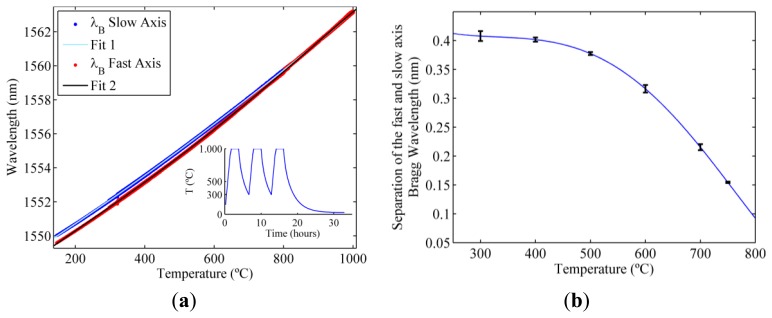
(**a**) Wavelength shift with temperature of the slow and fast axis Bragg resonances; (**b**) Separation between the slow and fast axis Bragg resonances.

The wavelength shift of the two resonances in the RFBG is characterized using temperature cycles. These characterization cycles use three concatenated temperature cycles that follows the same heating scheme of the regeneration process previously described. Nonetheless, in contrast with the regeneration process, between two consecutive temperature cycles the temperature is left to drop only down to 300 °C avoiding the long period of time needed to cool down the oven to ambient temperature. For temperatures higher than 800 °C the birefringence is smaller and is no longer possible to distinguish the two resonances with the instrumentation used and only one resonance is observed. The results and the fitting with a fifth order polynomial are shown in Figure 2a. Figure 2b shows the separation between the slow and fast axis Bragg resonances and the error between the fitting and the experimental data.

## 3. Interrogation Setup

**Figure 3.**
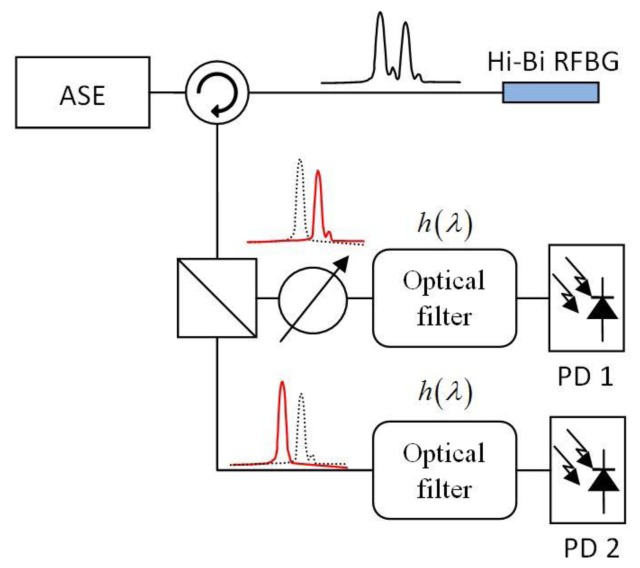
Proposed interrogation setup. ASE stands for Amplified Spontaneous Emission and PD for Photodiode.

In order to reduce the cost of the sensor interrogation, the scheme that is shown in Figure 3 is proposed. The light from a non-polarized optical light source illuminates the RFBG. The reflected signal is then divided into the two polarizations. A variable attenuator can be used to compensate possible differences in the optical power between the two polarizations. Each of the polarizations passes through an optical filter that converts the wavelength shift into power changes that is measured with a photodiode. Finally, the outputs of the two photodiodes are subtracted to determine the temperature variation. Optical power fluctuations are compensated due to the differential detection scheme on condition that optical variations affect the two polarizations at the same time. The use of the optical fibre filters results in a significant cost reduction compared with tunable lasers and tunable filters within the actual commercial interrogation units.

The response of the interrogation scheme can be tuned designing the optical spectrum of the optical filters. Standard optical edge filters can be used. In this case the output of the interrogation setup would show a non-lineal response, according to the temperature characterization, requiring a later electronic compensation. Alternately, signal processing can be performed optically designing and implementing the optical edge filter [11]. In an ideal situation, the two filters are the same and the output of the interrogation setup has a linear dependence with temperature. Since the optical filter is the same for the two polarizations the optical spectrum of the optical filter can be determined by a recursive relationship:

(1) $$\Delta P=h(\lambda_{1})-h(\lambda_{2})=A\cdot T+B$$

(2) $$h(\lambda_{1})=h(\lambda_{2})+A\cdot T+B$$

where Δ*P* is the optical power difference measured in the photodiodes, *h*(*λ*) is the spectrum of the optical filter where *λ*_1_ and *λ*_2_ are the wavelengths of the Bragg resonances for the fast and slow axis. *A* and *B* are the constants that define the linear dependence with temperature. The value of *A* is limited by the maximum attenuation of the optical filter and *B* can be obtained from the temperature *T_M_* where the two Bragg resonances match:

(3) $$B=-A\cdot T_{M}$$

**Figure 4.**
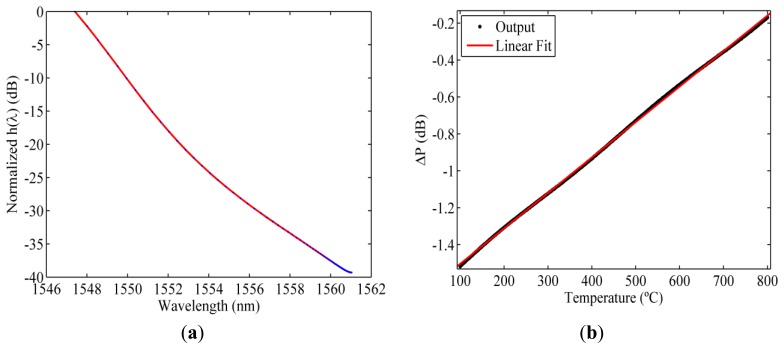
(**a**) Optical spectrum of the ideal filter; (**b**) Simulated output of the proposed interrogation setup using the ideal filter.

Figure 4a shows the optical spectrum of the ideal filter obtained using the recursive method and the temperature characterization of the RFBGs. Figure 4b represents the simulated output of the interrogation setup when the ideal filter is used. As can be noticed, the output has a linear dependence with temperature, as expected. Nonetheless, the slope of the ideal filter is limited by the high wavelength shift of the RFBG reducing the resolution of the interrogation setup. This limitation can be solved allowing the output of the interrogation setup be non-lineal.

## 4. Modal Interferometers

**Figure 5.**
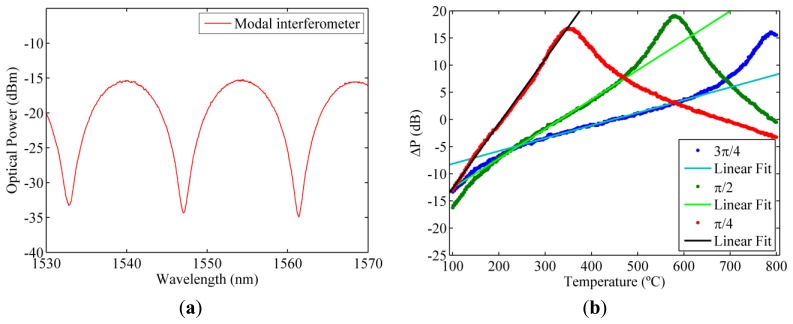
(**a**) Optical spectrum of a modal interferometer with a period of 14.3 nm; (**b**) Simulated output of the proposed interrogation setup using modal interferometers with relative phase differences.

The use of highly tunable filters permits the use of the same optical filter for both polarizations. Tuning one of the optical filters allows one to change the output of the interrogation setup improving the sensitivity of the interrogation setup. Using the interrogation setup proposed previously, the use of modal interferometers as optical filters is discussed. The spectra of modal interferometers show a periodic response that can be modelled as a sinusoidal response [12]. The period of the sinusoid can be easily selected during the fabrication of the modal interferometer and strain or temperature can be used to shift the spectral response. Figure 5a shows the spectrum of a modal interferometer with a period of 14.3 nm, where the period of the modal interferometer has been selected to be larger than the wavelength shift of the RFBG for high temperatures.

The output of the interrogation setup is simulated using the measured optical spectra of the modal interferometer and the RFBG. As a result of the small separation of the two Bragg resonances the performance of the interrogation setup can be improved introducing a wavelength shift between the two optical filters. This wavelength shift is equivalent to introduce a phase shift in the sinusoidal optical spectrum of the modal interferometers. It is worth to mention that, because of the periodic response of the modal interferometers, the unambiguous range is reduced at the same time. Figure 5b shows the effect of the relative phase difference induced between the two optical filters.

Using the modal interferometers the linear range obtained is limited by the period of the modal interferometer and the relative phase difference. The sensitivity, which is determined by the slope of the output of the interrogation setup, depends on the relative phase difference. The linear range can also be tuned applying the same wavelength shift to both optical filters.

## 5. Conclusions

The fabrication and characterization of regenerated fiber Bragg gratings in high-birefringence optical fiber for use in high-temperature applications has been shown. The optical spectrum shows two Bragg resonances for the slow and fast polarizations. The separation of the two Bragg resonances can be used to reduce the cost of an interrogation unit using optical filters to convert the wavelength shifts into optical power changes. The spectral response of the optical filter has been studied. The ideal filter which linearizes the output of the interrogation setup has been obtained but the sensitivity is limited by the large wavelength shift of the RFBG and the maximum attenuation of the filter. To avoid the limitations of the ideal optical filter the output of the interrogation setup is studied using modal interferometers. Modal interferometers are highly tunable devices which optical spectrum can be easily shifted by temperature or strain. Due to the periodic response of the modal interferometers the output of the interrogation setup is also periodic with a quasi linear response in a limited range. The range and slope of the interrogation setup can be tuned by modifying the phase difference between the modal interferometers.
